# Development and testing of a high-risk behavior scale for swimming among Chinese adolescents

**DOI:** 10.3389/fpsyg.2025.1655110

**Published:** 2026-01-12

**Authors:** Chao Lu, Jiaxin Shi, Hui Zhang

**Affiliations:** 1 Zhixing College of Hubei University, Wuhan, Hubei, China; 2 School of Physical Education, Hubei Minzu University Enshi, Hubei, China

**Keywords:** adolescents, reliability, scale development, swimming risky behavior, validity

## Abstract

**Background:**

Teenagers are the main victims of drowning, and high-risk behavior is the main cause of drowning. However, there are no reliable and valid instruments for measuring these behaviors in China.

**Objective:**

This study aimed to provide a measurement tool for measuring the high-risk behavior of swimming among adolescents in China. The adolescent swimming risk behavior scale was developed and tested for reliability and validity.

**Participants and setting:**

This study used stratified sampling to ensure sample representativeness. Stratification variables included educational level (elementary school, secondary school) and geographical region (areas with differing levels of economic development), thereby capturing key differences in age maturity and social environment. Among schools selected based on the aforementioned stratification variables, questionnaires were administered to eligible students (aged 10–19 years old, capable of swimming continuously for over 25 m).

**Methods:**

This study assessed the internal consistency, factor structure (i.e., exploratory and validation factor analyses), and convergent and discriminant validity of the adolescent swimming risk behavior scale.

**Results:**

The findings suggest that the scale is a relatively reliable measure for assessing high-risk behavior in adolescent swimmers. Exploratory and validation factor analyses confirmed the four-factor structure, confirming the 20-item version of the scale, which obtained good internal consistency reliability. The internal consistency coefficient, Cronbach’s *α*, for the formal scale ranged from 0.764 to 0.869, with the total scale Cronbach’s *α* value being 0.859.

**Conclusion:**

The adolescent swimming risky Behavior Scale has high reliability and validity and is suitable for measuring adolescent swimming risky behavior in China.

## 1 Introduction

Swimming high-risk behavior is a key causative factor leading to adolescent drowning, which refers to risky behaviors with the risk of self-injury or other injuries performed by an individual or a group of individuals in an aquatic environment (World Health Organization, 2014). Data from the National Health Commission (NHC) show that approximately 56,000 adolescents lose their lives to drowning each year in China, and the loss of individual lives and socio-economic burden have become a major challenge in public health (UNICEF, 2009). For this reason, as of 2025, the Ministry of Education has issued special warning notices for 14 consecutive years to prevent possible swimming injuries (The State Council of the People’s Republic of China, 2025).

Extant research has identified several key factors that elevate drowning risk among adolescents. These commonly include a lack of supervision, overestimation of swimming ability, risk-taking tendencies, peer pressure, and swimming in unsafe environments. Understanding these foundational risk factors is crucial, as they directly manifest themselves in specific, observable high-risk behaviors.

Measurement of high-risk behaviors in swimming has gone through three phases: the initial exploratory phase (2006–2010), when Moran (2006) in New Zealand developed the first waterside high-risk behaviors scale in 2006, which assessed the risks associated with unsupervised solo swimming, ignoring safety instructions, and other supervisory deficits through 10 entries. In 2008, McCool et al. (2008) further refined the original scale with three entries specifically assessing adult swimming risk behaviors; later expanded by Morrongiello et al. (2010) in Canada to form a special measurement tool for adolescents’ at-risk swimming behaviors. The theoretical deepening phase (2012–2017) (Xia et al., 2012) achieved a breakthrough in measurement dimensions with the localized scale constructed based on the theory of knowing, believing, and acting. Petrass and Blitvich (2014) proposed the behavioral dichotomy to differentiate between misbehaviors and lapses in high-risk behaviors for the first time to promote the innovation of the theoretical framework, and Zhang et al. (2017) formed 10 subcategories of high-risk behaviors affecting students by rooting the coding of the interviews and further refined the Erroneous and lapsed behaviors further refined, and for the first inclusion of bad peer infractions in environmental factors occurred in; the systematic refinement phase (2019–2024), in which the peer influence model established by Luo et al. (2019) and the infraction subtypes revealed by the subsequent studies (Taiwo et al., 2024; Vannucci et al., 2020) together improved the behavioral taxonomy system.

Currently, the measurement tools for studying adolescents’ swimming high-risk behaviors are facing a double challenge: first, adolescents have always been the focus of the WHO initiative to intervene in high-risk groups, and swimming drowning accidents among adolescents in China are frequent, so a set of measurement tools suitable for adolescents’ swimming high-risk behaviors in China is urgently needed; and second, there is the lack of discriminant validity of the Swimming High-Risk Behavior Scale in the behavioral motivation dimensions (e.g., risk-taking tendency vs. aggressive violations). Therefore, the research group comprehensively considered the psychological and behavioral characteristics of adolescents in China and conducted interviews and surveys on adolescent swimming risky behaviors to develop a set of scientific and effective scales for adolescent swimming risky behaviors, which is of great significance for measuring adolescent swimming risky behaviors, identifying risky groups, and intervening accurately in advance.

## 2 Research procedure

The initial project library primarily originated from a qualitative study using grounded theory methodology, which constructed a model of factors influencing students’ high-risk behaviors in aquatic environments (Xia et al., 2012; Zhang et al., 2017). Subsequently, insights from systematic literature reviews and expert interviews supplemented and refined this foundational framework, ultimately yielding preliminary scale items for assessing high-risk swimming behaviors among adolescents (Koon et al., 2021; Petrass and Blitvich, 2014; Xia et al., 2018). The content validity test was carried out through the discussion of the research group and the consultation of experts (a total of five experts were invited to participate in the questionnaire completion process across the fields of water safety education, swimming rescue training, swimming instruction, physical education, and training). Among them, three are professors and doctoral advisors, one is an associate professor and master’s advisor, and one is a lecturer, focusing on the readability, meaning, logic, wording, and relevance of the scale entries, etc. The pre-testing scale of adolescent swimming high-risk behaviors was formed after repeated revisions and refinements, and the pre-survey was carried out after that. After that, a pre-survey was conducted, and the recovered data were analyzed through item analysis, exploratory factor analysis, and deletion of questions to form a formal scale; a formal test was conducted, and the formal scale was compiled after validation factor analysis and reliability and validity tests.

## 3 Pre-test of the scale

### 3.1 Pre-test entries of the scale

Based on the results of the interviews and relevant literature, and combining China’s waterside environment, adolescent psychology, and behavioral habits, the scale was repeatedly refined and modified: (1) to ensure that the overall structure and logic of the scale would not be damaged, (2) to follow the principle of singularity to avoid composite questions when modifying the questions to make sure that the respondents could clearly and accurately understand the intent of the questions, and (3) to ensure the scale’s integrity, a 28-item pretest version was developed by synthesizing the Knowledge-Attitude-Practice theory and the Rational Behavior Theory, identifying four core factors: “Wrong Behavior,” “Mistake Behavior,” “General Violations,” and “Aggressive Violations.” Wrong behavior entails intentional but incorrect actions stemming from deficient knowledge or biased risk perception (e.g., misjudging one’s swimming ability), whereas mistake behavior involves unintentional execution failures where a correct action plan goes awry due to attentional lapses or skill-based errors (e.g., slipping unintentionally). This clarifies that wrong behavior originates in cognitive judgment, while mistake behavior arises from failures in physical execution. For intentional violations, general violations are driven by social norms such as peer influence, and aggressive violations by internal attitudes, such as risk-seeking. This framework ensures discriminant validity by systematically categorizing behaviors from unintentional cognitive and execution errors to socially and internally motivated intentional violations (Bozzini et al., 2020; Huh and Reif, 2020; Park, 2000; Lu, 2025). All entries were scored on a 5-point Likert scale, where subjects were asked to rate each indicator on a scale of 1–5, with 1 being very poor and 5 being very good.

### 3.2 Pre-survey and analysis

#### 3.2.1 Subjects of the survey

Given that the WHO defines the age of adolescents as 10–19 years old (World Health Organization, 2017) and that students in this age group account for a very high proportion of drowning accidents (Public Opinion Data Center of People’s Daily Online, 2022). This study used stratified sampling to ensure sample representativeness. Stratification variables included educational stage (elementary school, secondary school) and geographical region (areas with varying levels of economic development), thereby capturing key differences in age maturity and social environment. Preliminary surveys were conducted among eligible students (aged 10–19 years old, capable of swimming continuously for over 25 m) in schools selected based on the aforementioned stratification variables. A total of 587 questionnaires were distributed, and 550 were returned. After data cleaning, 523 valid questionnaires were obtained, yielding a response rate of 89.1%. The sample comprised 275 males (52.6%) and 248 females (47.4%).

#### 3.2.2 Item analysis

First, based on the pretest data (*n* = 523), a critical ratio (CR) analysis was conducted on the 28 items using SPSS 27.0. The total scores of the pretest sample data were sorted in descending order, and the critical values for the top and bottom 27% groups were calculated. Independent samples *t*-tests were then performed on each item within the high and low score groups. Results revealed significant differences between high- and low-scoring groups for all 28 items (*t* > 3, *p* < 0.05), indicating good discriminative validity suitable for further analysis. Subsequently, items exhibiting non-significant correlations with total scores (*p* > 0.05) or low correlations (*R* < 0.4) were excluded. Four items failing these criteria were identified and removed: Item 2 (when discovering someone drowning, one should shout for help before entering the water to rescue them), Item 17 (sometimes arranging to swim in unfamiliar waters with friends is more exciting), Item 23 (when swimming, if someone blocks your path, it is acceptable to pull them aside to avoid collision), and Item 28 (it is fun to ride on someone’s back while swimming).

#### 3.2.3 Exploratory factor analysis

According to the pretest data (*n* = 523), the sample feasibility analysis was conducted first, and the sampling suitability measure (KMO) value of the total sample was 0.887, which indicated that the sample data were well suited for factor analysis, and the sig value of the Bartlett’s test of sphericity was 0.000, which indicated that there was a sufficient correlation between the variables to be suitable for factor analysis (Quintero Ordóñez et al., 2021; Hai-ming L, 2009; Table 1).

**Table 1:** KMO and Bartlett test for pretest data.

Test item		Value
KMO quantity of sampling fitness		0.889
Bartlett’s test of sphericity	Approximate chi-square	7,613.042
	Degree of freedom	190
	Significance	0.000

**Figure 1:**
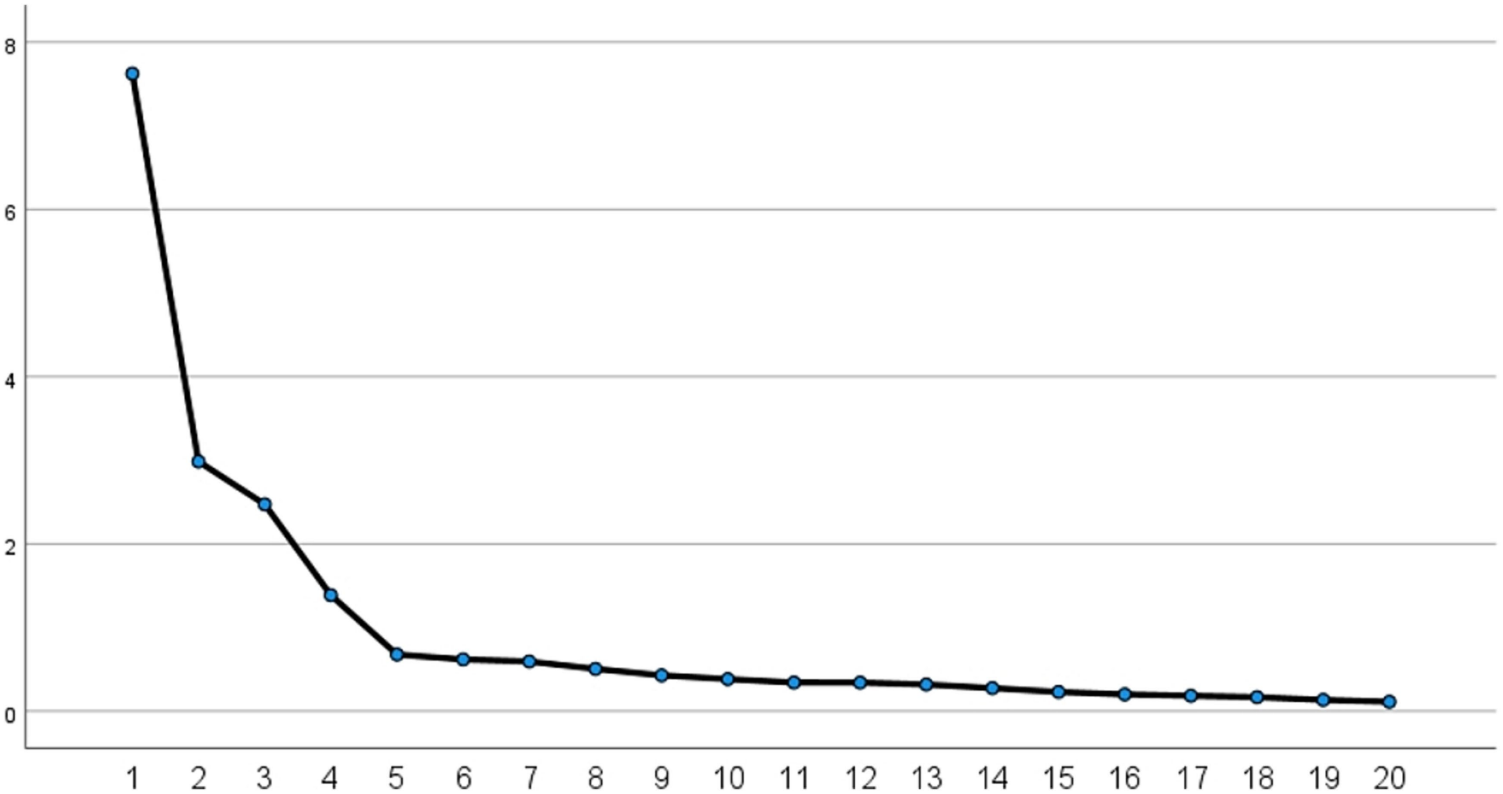
Scree plot of the preliminary scale for high-risk behaviors in adolescent swimming (*n* = 523).

The factors were extracted by principal component analysis, and the optimal oblique intersection method (Kappa = 4) was used for exploratory factor analysis, and the factors were extracted by the criterion of eigenvalue greater than 1, and a total of 4 factors were extracted. The results showed that the cumulative variance contribution of the four-factor model was 72.362% (38.121, 14.931, 12.372, and 6.939%), and the curve flattened after the fourth factor of the gravel plot (Figure 1), and the model construction was reasonable. After gradually deleting the entries with loadings less than 0.5 (Item 3: swimming in water bodies with health hazards; Item 7: when your feet get tangled in aquatic plants while swimming, you must struggle vigorously to free yourself; Item 13: swimming on an empty stomach makes it easier to lose weight; Item 21: wearing swimming safety equipment allows you to swim in areas with warning signs), 20 entries were finally retained to form a four-dimensional structure: “Wrong Behavior,” “Mistake Behavior,” “General Violation” and “Aggressive Violation,” and the entries converged well on the four factors (Table 2).

**Table 2:** Exploratory factor analysis of the preliminary scale for high-risk behaviors in adolescent swimming (*n* = 523).

Item	Factor			
	Wrong behavior	Mistake behavior	General violation	Aggressive violation
Q1 When a companion is drowning, everyone can hold hands and work together to rescue the companion.	0.929			
Q6 Swimming and relaxing in the water after a long time of intense exercise is very exciting.	0.835			
Q5 I can swim for as long as I want to swim.	0.794			
Q4 It’s okay to swim directly after arriving at the pool.	0.749			
Q14 Wearing too long or ill-fitting swimsuits and trunks when swimming.		0.917		
Q11 People who can swim are sure they will not drown.		0.850		
Q8 You can rest in the water if you are tired from swimming for a long time but do not want to go ashore yet.		0.847		
Q9 It is safe to play in the pond as long as you do not go into the water.		0.822		
Q12 before swimming must be warmed up, into the water to stretch a little, to the chest and back to pat the water on it.		0.795		
Q10 You can go into deep water to play after you can swim.		0.695		
Q15 You can dive in pools that are guarded by lifeguards.			0.929	
Q16Swimming in unsecured wild waters.			0.904	
Q19 It is okay to go to open water to catch fish, touch snails, or go boating.			0.830	
Q20You can try to learn to swim on your own in water that is not too deep.			0.825	
Q18 Swimming without an adult.			0.676	
Q27 When someone is afraid to go into the water, you can give him a hand and push him into the water.				0.930
Q22You can play and fool around in the water or around the pool with your peers.				0.923
Q26 Taking away a classmate’s flotation device and letting him swim on his own so that he can learn to swim as soon as possible.				0.857
Q25 Splash water on a swimming partner.				0.795
Q24 When swimming in a pool, it is okay to stop and rest or swim back and forth in the lane that others are swimming in.				0.756
Characteristic value	7.624	2.986	2.474	1.388
Contribution (%)	38.121	14.931	12.372	6.939

The extraction method is Principal Component Analysis; the rotation method is Kaiser Normalized Optimal Oblique Crossing; the rotation has converged after 7 iterations.

## 4 Formal survey of the scale

### 4.1 Survey objects

Formal measurements were taken in selected primary and secondary schools in China (selection criteria: school students who can use any stroke to swim continuously for more than 25 m); a total of 1,500 copies of the scale was issued, 1,452 copies of the scale were recovered, excluding the invalid scale. A total of 1,303 copies of the valid scale were obtained, and the validity of the recovery rate was 86.8%. Among them, there were 684 boys and 619 girls, 549 aged 10–13 years old, 481 aged 13–16 years old, and 273 aged 16–19 years old.

### 4.2 Validated factor analysis

Validation factor analysis was implemented using AMOS 27.0 on the data from formal measurement subjects (*N* = 1,303) to test the rationality and validity of the model. As shown in Table 3, the *χ*^2^/df value was 3.672, indicating that the model fit to the data was acceptable (Collier, 2020); THE CFI value was 0.938, and the IFI, TLI, and NFI are all over 0.9, indicating that the model has an excellent fit; the RMSEA value is 0.045, and the RMR value is 0.048, which further confirms that the model matches well with the actual data (Cheung and Rensvold, 2002; Walker and Smith, 2017; Table 3).

**Table 3:** Confirmatory factor analysis of the scale for high-risk behaviors in adolescent swimming (*n* = 1,303).

Fitting index	*χ*^2^/df	CFI	IFI	TLI	NFI	RMR	RMSEA
4-dimensional model	3.672	0.938	0.938	0.957	0.952	0.048	0.045

**Figure 2:**
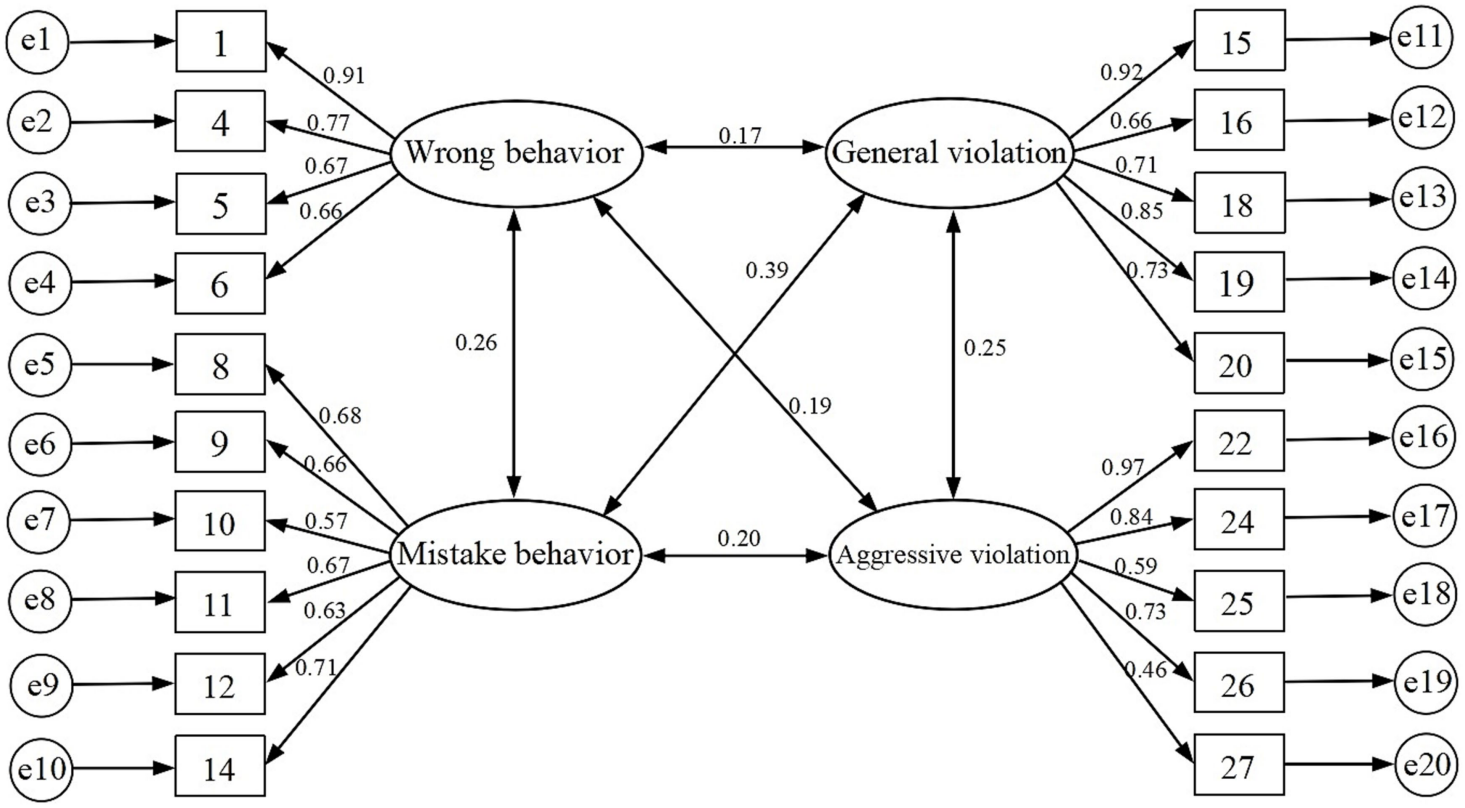
Four-factor structural model diagram of the scale for high-risk behaviors in adolescent swimming (*n* = 1,303).

The structural equation model diagram (Figure 2) shows factor loadings all exceeding 0.4, indicating that the observed variables are appropriately set, the model fits well, and the scale possesses good construct validity. The correlation between “Wrong Behavior” and “Mistake Behavior” in the model (*r* = 0.26), though weak, is significantly higher than their respective correlations with “general violations” and “aggressive violations.” This subtle yet verifiable association precisely corroborates the theoretical hypothesis that both concepts share a tenuous link due to their inherent “non-intentional” components. More significantly, the overall correlation structure indicates that “mistake” and “violations,” sharing the characteristic of “knowing yet acting,” exhibit generally stronger associations than their distinct root causes with “Wrong Behavior.” This fully demonstrates that the scale effectively distinguishes between the more fundamental behavioral drivers of “knowledge deficiency” and “motivational deviation.” Ultimately, this study developed the Adolescent Swimming Risk Behavior Scale, comprising 20 items across four primary dimensions.

### 4.3 Scale reliability analysis

The internal consistency coefficient (Cronbach’s *α* coefficient) was applied to test the internal consistency of the formal measurement data (*n* = 1,303) of the scale. It was found (Table 4) that Cronbach’s *α* = 0.859 for the total scale of adolescent swimming high-risk behaviors, indicating that the scale as a whole has high reliability; Cronbach’s *α* for the subscales of mistake behavior, general violation, and aggressive violation exceeded 0.8, and the Cronbach’s *α* for wrong behavior was 0.764, indicating that the reliability of each subscale was good (Tavakol and Dennick, 2011).

**Table 4:** Reliability analysis results of the scale for high-risk behaviors in adolescent swimming (*n* = 1,303).

Dimension	Cronbach’s *α*	Number of terms
Wrong behavior	0.764	4
Mistake behavior	0.859	6
General violation	0.869	5
Aggressive violation	0.804	5
Youth swimming risk behavior scale	0.859	20

### 4.4 Scale validity analysis

To ensure the content validity of the scale, this study first invited experts in the fields of water safety education, swimming rescue, and training theory to assess the content suitability of the scale entries and then verified the convergent validity and reliability by calculating the AVE and CR. The results showed (Table 5) that the AVE values of misbehavior, general violations, and aggressive violations were above the standard threshold of 0.5, and the AVE value of misbehavior was 0.4307, which was in the acceptable range (0.36–0.5) (Kline, 2016). For emerging behavioral scales measuring complex concepts, this phenomenon is well-documented. We nevertheless retain this dimension due to its critical theoretical significance in reflecting cognitive bias risks. Therefore, although the AVE values indicate that future refinement of item design is warranted, the reliability, distinctiveness, and theoretical value of this dimension sufficiently justify its inclusion; the Cronbach coefficients of all dimensions and the CR values were greater than the recommended standard of 0.6, which indicated that the internal consistency of the scale was good. According to the existing research standards, the above indicators confirm that the scale has reliable convergent validity and reliability and can effectively support the testing of research hypotheses and the derivation of conclusions (dos Santos and Cirillo, 2021).

**Table 5:** Validity analysis results of the scale for high-risk behaviors in adolescent swimming (*n* = 1,303).

Latent variables	CR	AVE
Wrong behavior	0.842	0.576
Mistake behavior	0.819	0.431
General violation	0.898	0.640
Aggressive violation	0.860	0.563

## 5 Discussion

The construction of the four-dimensional structure for measuring adolescent swimming high-risk behavior, comprising wrong behavior, mistake behavior, general violation, and aggressive violation, represents the core theoretical innovation of this study. While grounded in established frameworks such as the theory of knowing, believing, and acting, this model moves beyond them by proposing a nuanced taxonomy that differentiates risk behaviors based on their underlying mechanisms: from unintentional cognitive errors (wrong behavior), which are linked to adolescents’ underestimation of risk related to the incomplete development of the prefrontal lobe (Pinson et al., 2021) and skill-based lapses (Mistake Behavior) that occur at a higher rate under stress, particularly in adolescents compared to adults (Hu et al., 2020; Causer et al., 2021), and are often due to a lack of ability or chance factors such as cramp handling (Moran and Gilmore, 2018; Moran et al., 2018), to intentional but non-malicious breaches (general violation), which are prevalent and significantly influenced by parental absence and the presence of peers (Ismail et al., 2021; Public Opinion Data Center of People’s Daily Online, 2022), and willfully harmful acts (aggressive violation), which are strongly predicted by externalizing problems and sensation-seeking (Olivier et al., 2020; Liao et al., 2021; de Ruijter et al., 2023), a trait that also amplifies the influence of undesirable peers (Luo et al., 2019). This granular classification addresses a critical gap in existing scales, which often lack such differentiation, and provides a more precise theoretical lens through which to understand how risky behaviors originate and escalate, including the neurodevelopmental perspective that prefrontal lobe development lag leads to risky decision-making bias (Hamilton et al., 2024).

The practical value of this four-dimensional framework lies in its direct applicability to targeted intervention. Distinguishing between error-based and violation-based behaviors enables the development of stratified prevention strategies. For instance, wrong and mistake behaviors may be best addressed through safety knowledge education and skill-based training, as they are tied to cognitive bias and operational error. In contrast, general and aggressive violations, which are strongly associated with social context, peer influence, and sensation-seeking, require interventions that modify subjective norms and enhance perceived behavioral control, an approach supported by the Theory of Planned Behavior’s efficacy in explaining behavioral intention (Cao et al., 2014). Moreover, the finding that prolonged uncorrected errors may evolve into habitual violations or even aggressive violations underscores the need for early and type-specific responses.

This study thus validates and extends existing theoretical research by offering a structured behavioral continuum that connects cognitive, behavioral, and social-ecological factors. It shifts the focus from generic drowning prevention to a differentiated intervention model, informed by the social-ecological model’s emphasis on the interaction of microsystem, mesosystem, and macrosystem factors (Van Wormer and Besthorn, 2017), in which families, schools, and policymakers can collaborate to address specific risk pathways with greater precision. In doing so, it establishes a new foundation for both measuring and mitigating swimming-related risks among adolescents in a theory-informed and empirically actionable manner.

## 6 Conclusion and recommendations

### 6.1 Conclusion

The factor structure of the Chinese Adolescent Swimming Risk Behavior Scale revealed by the analysis warrants particular attention. The scale identified four distinct dimensions: wrong behaviors, error-prone behaviors, general rule-breaking behaviors, and aggressive rule-breaking behaviors. This indicates that adolescent swimming risk is not a single construct but comprises conceptually distinct categories. Compared to unidimensional models, this finding provides a more nuanced framework for understanding risk behaviors. Crucially, the evolutionary trajectory implied by the dimensional labels from unintentional errors to deliberate aggression reflects an inherent continuum of intent and severity. This aligns with and extends existing social psychological theories of risk behavior. This structural validation constitutes the core theoretical contribution of this study.

### 6.2 Recommendations

(1) The scope and sample of this study may be limited. Findings are based on a specific geographic region, limited sample size, or particular demographic group, which may affect the generalizability of results to broader adolescent populations. Future research should focus on collecting data across a wider range and multiple levels (e.g., different regions, socioeconomic backgrounds, and swimming skill levels) to gain a more accurate and comprehensive understanding of the current situation.
(2) The measurement tools and methods used may not fully capture the complexity of high-risk swimming behaviors. Future research could benefit from adopting mixed methods, combining surveys with observational studies, in-depth interviews, or incident report analyses to triangulate findings and identify more detailed risk factors.
(3) This study primarily focused on identifying risk factors without evaluating the effectiveness of specific interventions. The proposed future data collection would provide a robust empirical foundation for developing targeted safety education strategies, preventive measures, and emergency plans. Subsequent research should concentrate on developing, implementing, and rigorously evaluating the efficacy of these interventions through randomized controlled trials or longitudinal studies.

## Data Availability

The raw data supporting the conclusions of this article will be made available by the authors, without undue reservation.
